# Results of interventional treatment of peripheral slow-flow malformations

**DOI:** 10.1186/s42155-023-00352-3

**Published:** 2023-02-10

**Authors:** Jens Altenbernd, Felix Kutta, Michael Forsting, Jens Theysohn, Stefan Rohde

**Affiliations:** 1 Institute of Diagnostic and Interventional Radiology and Neuroradiology, University Hospital Essen, Hufelandstr. 22, 45122 Essen, Germany; 2 Radiology and Neuroradiology, Klinikum Dortmund gGmbH, Dortmund, Germany

**Keywords:** Peripheral malformations, Slow flow, Intervention, Embolization

## Abstract

**Background:**

In recent years sclerotherapy has increasingly become the treatment of choice for peripheral slow-flow malformations. However, the long-term effectiveness of sclerotherapy is still a matter of debate, especially when it comes to new sclerosing agents like polidocanol. This study aims at gathering further information concerning its long-term effectiveness and safety.

**Results:**

Most patients reported a reduction of symptoms which include pain (57,7%), swelling (65,4%) and functional impairment (60%). Cosmetic complaints were less likely to be reduced by sclerotherapy (44,4%). In most cases a relief of symptoms was stable for many years, especially after several consecutive treatment sessions. Complication rates were comparably low, with only 2 patients requiring additional treatment at hospital and no lasting damages. (…) (7) Most patients (70,9%) were at least partially satisfied with the treatment. Satisfaction was closely linked to a partial or complete relief of symptoms (*p* = 0.001).

**Conclusion:**

Sclerotherapy is a promising way of treating slow-flow-malformations. Polidocanol has proved to be a save sclerosing agent. The reduction of major symptoms was substantial in most cases and lasted for many years.

## Introduction

The treatment of slow-flow malformations (sfM) has continuously been improved during the last decades. For a long time, surgery was the only available treatment option alongside conservative therapy. However, long-term symptom reduction was only achieved in a minority of patients. Since the late 1980s, interventional procedures (sclerotherapy) have increasingly found their way into everyday clinical practice. While a short-term symptom reduction has already been demonstrated in early studies (Yakes et al. 1989), more recent studies also indicate long-term effects (Linden et al. 2009). That is one reason why sclerotherapy is increasingly preferred to surgery and considered the gold standard in the treatment of venous malformations.

This study aims at gathering information concerning the long-term effectiveness and safety of sclerotherapy. Moreover we focus on overall patient satisfaction with the procedure.

## Material and methods

We included all patients with periphereal sfM that had been treated in Dortmund between 2013 and 2021. All patients concerned were treated with polidocanol. However, persons with high-flow malformations (hfM) or central sfMs were excluded. Furthermore, patients with primary vascular surgery therapy were not considered in order to allow for an unbiased evaluation of the effectiveness of Sclerotherapy (Gulsen et al. 2011).

(…) The monotonic relationship between two variables is quantified on the basis of Spearman's rank coefficient. A comparison between different subgroups with regard to possible influencing factors on patient satisfaction and the treatment result, was carried out using Fisher's exact test (two-tailed). As is usual in comparable studies, a significance level of 5% was adopted.

After agreeing to participate in our study, patients received a questionaire which focused on main symptoms of sfM before treatment, the scale of symptom relief after treatment, the duration of symptom relief and complications. Moreover we used the questionaire to assess overall satisfacion with sclerotherapy (Nevesny et al. 2021).

When evaluating the questionnaire, percentages always refer to the number of patients who answered the respective question, not the total number of all participants. The data was initially compiled in Microsoft Excel© (version 16.60) and then evaluated in the statistics program R (version 4.1.3).

Our study was approved by the Ethics Committee of the University of Duisburg-Essen (reference: 20–9764-BO) and carried out in accordance with the Declaration of Helsinki.

## Results

A total of 41 patients were eligible for our study, but the current address of five patients could not be determined. One patient declined to participate in the study. Seven patients did not respond to our repeated requests throughout the data collection period. 28 patients (68.2%) agreed to participate and answered our questionnaire. The volume before treatment was estimated on the basis of magnetic resonance images (MRI) in 21 cases. For 15 patients (36.5%), volume before and after the intervention could be compared. 12 patients (29.2%) had an appointment for the clinical follow-up during the ongoing study. In this case, additional clinical parameters (swelling, pain etc.) could be recorded.

In our patient cohort, the youngest person was 7 y at the time of the first intervention, and the oldest 64 y. The mean age (*n* = 28) at the first intervention was around 33 y (median = 34 y). From 0 to 60 y there was almost an equal distribution, which is why the variance was very large (SD = 17.9).

MRI is the diagnostic tool of choice, especially for imaging deep malformations. Therefore, almost all patients (92.9%, *n* = 26) received this imaging for further therapy planning. Ultrasound (US) was also used as a supplement, particularly in the case of superficial sfM (35.7%, *n* = 10). Computed tomography (CT) was only used in a minority of cases (28.6%, *n* = 8) and was usually supplemented by an MRI examination.

HfM and sfM were distinguished by means of US and MRI-Imaging. Normally typical features of VM (slow enhancement, Pheloboliths etc.) were sufficient to rule out a hfM. However in some cases DSA was necessary to distinguish the two (Spence et al. 2011).

All in all, this study includes 25 patients with VM and 3 patients with LM. Clinical examination and US were normally sufficient to differentiate these two common types of sfM (Ali and Mitchell 2017).

During the actual intervention, an ultrasound was always (*n* = 28) performed in order to position the puncture needle correctly. Superficial sfM were punctured by using butterflies. For deep sfM we normally used 20G needles. Then contrast agents were injected to image size, structure and venous drainage of the respective malformation. In case there were multiple compartments that had to be treated, we punctured these separately. We then injected polidocanol while compressing draining veins. Imaging in roadmap technique was used to demonstrate the distribution of polidocanol within the malformation. All compartments were treated accordingly, while we always respected the maximum dose for polidocanol (Odeyinde et al. 2013).

After treatment, compression was applied according to the location of the respective sfM. SfM of the limps were compressed by elastic bandages for at least 10 days. Head and neck sfM were compressed manually for 10–15 min immediately after treatment. If possible, we then used compression bandages for around 24 h. (Schmidt et al. 2022).

The length of stay depended on individual complaints after treatmend and was usually limited to one day. In some cases, patients stayed at our hospital for up to 3 days after treatment (Schmidt et al. 2021).

As in previous studies, we assumed ellipsoid shape to estimate the volume (see for instance Linden et al.). MRI images were used to measure the size of each sfM (Aronniemi et al. 2016). All in all, we calculated the initial volume in 21 patients. For the remaining patients we either did not have sufficient imaging material to analyse or the sfM were too diffuse to allow for volume approximation (Clemens et al. 2017).

The smallest malformation had a volume of only 7 ml, while four malformations took up more than one liter (maximum = 3458 ml). The average volume was about 641 ml (median = 219 ml) (…) (Behravesh et al. 2016).

In our study, the majority of cases (60%, *n* = 15) were diffuse sfMs. Skin and subcutaneous fat tissue (60.7%, *n* = 17) and muscles (71.4%, *n* = 20) were affected particularly frequently. Bone involvement was less common (21.4%, *n* = 6). A direct infiltration of internal organs could not be demonstrated in imaging. However, one sfM each infiltrated the mucous membranes in the area of ​​the genitals and the larynx.

Most (60%, *n* = 15) VM showed slow drainage, in two cases a reliable assessment was not possible. The remaining VM (32%, *n* = 8) were characterized by a fast flow.

On average, 1.5 interventions were carried out (maximum = 4). Overall, this study includes 43 interventions on 28 patients over a period of about seven years. The majority of those affected (60.7%, *n* = 17) were treated exclusively in our departement. However, a total of eleven patients (39.3%) went to another clinic beforehand. (…) This explains why some patients indicated significantly more interventions when answering the questionnaire. All interventions were based on the injection of a Polidocano-solution. The concentration used (3%) is applied to sfMs and large varices, while low concentrations (0.25–2%) be preferred in the treatment of spider vein varices.

As mentioned above the applied dose of polidocanol depended on the size of the respective sfM and was limited to the maximum dose (2 mg/kg BW). The Spearman rank correlation coefficient supports this and suggests a strong positive correlation between the initial volume of the respective sfM and the dose of polidocanol administered (rs = 0.597; *p* = 0.0243).

One focus of the questionnaire were typical symptoms of sfMs before the intervention. All participants (*n* = 28) answered this question. Multiple answers were allowed. Most patients complained about pain (92.9%, *n* = 26). Swelling in the area of ​​the lesion also occurred in 92.9% of all patients (*n* = 26). Both symptoms worsened episodically in most patients prior to treatment. A cosmetic impairment (64.3%, *n* = 18) was found especially regarding superficial malformations of the skin and subcutaneous tissue. Functional impairment (64.3%, *n* = 18) was particularly evident in malformations in the vicinity of joints. Rare symptoms (each *n* = 1) included e.g. Shortness of breath (malformation in the area of ​​the larynx), recurrent bleeding or venous congestion distal to the affected VM.

We also asked the patients to specify the extent of their pain more precisely. For this we used the classic pain scale from 0 (no pain) to 10 (worst imaginable pain). One patient did not answer this question (*n* = 27). We found that a majority of patients (*n* = 15) reported strong pain (> 5). Only two participants suffered from no pain at all (Marrocco-Trischitta et al. 2001).

### Symptom reduction through the intervention

A total of 21 patients reported alleviation of symptoms with regard to at least one of the above-mentioned symptoms. 19 patients commented on the number of interventions that were necessary for such a noticeable reduction in symptoms after all. 16 patients (84.7%) benefited from sclerotherapy after two procedures at the latest. In extreme cases, however, up to six sessions were necessary. As mentioned above, some of these were carried out in other clinics. A patient also reported that only the combination of sclerotherapy and vascular surgery showed the desired effect.

We also asked those patients who reported a relevant symptom reduction after sclerotherapy and had been treated more than 48 months ago (*n* = 11) how long this positive effect lasted. More than 60% (*n* = 7) of these patients still benefited from the treatment 48 months after the last intervention.

### Complications and complication management

The majority of patients (53.8%, *n* = 14) could not remember any complications during the procedure at all. Four patients (15.4%) reported more than one complication. Those affected complained mostly about swelling or pain in the area of ​​the puncture site. In order to prevent the serious complication of thrombosis, compression of the corresponding sfM following sclerotherapy is recommended. A total of 12 patients reported having performed compression after treatment, for instance by wearing compression socks. Some patients chose to extend compression for several years.

Fortunately, only two patients (18.9%) required additional hospitalizations due to sclerotherapy complications. For instance, one patient suffered from excessive swelling in the treated area (Nakamura et al. 2014). Two other patients required symptomatic treatment but were not re-admitted in our case. The remaining participants (63.3%, *n* = 7) were only affected so slightly that additional therapy was not required.

The subgroup analysis showed that patients with bone infiltration had more complications (66.7%, *n* = 4) than the rest of the cohort (40%, *n* = 8, *p* = 0.37). In patients with rapid venous drainage, complications occurred in 50% of cases (*n* = 4), while these were observed less frequently (40%, *n* = 6) in patients with slow drainage (*p* = 0.69). Patients with large and small VMs had similar complication rates. Complications were more common among those who had to be treated multiple times (66.7%, *n* = 6) than among those who came to Dortmund for only one session (35.3%, *n* = 6) (*p* = 0.22). (…).

### Patient follow-up

In the majority of patients (*n* = 10, 66.7%), a slight to moderate volume reduction was found when images before and after treatment were compared. In one patient we even observed a complete involution of the sfM after the intervention.

On average, the volume reduction was around 2.6%, with an interquartile range of around 35% (volume change -26.7% to + 9.3%).

### Patient satisfaction

A total of ten patients (41.7%) viewed the treatment in a completely positive way, and a further seven patients (29.2%) were at least partially satisfied with the procedure. Three patients (12.5%) were rather dissatisfied and four patients (16.7%) evaluated the treatment attempt as completely negative.

**Fig. 1:**
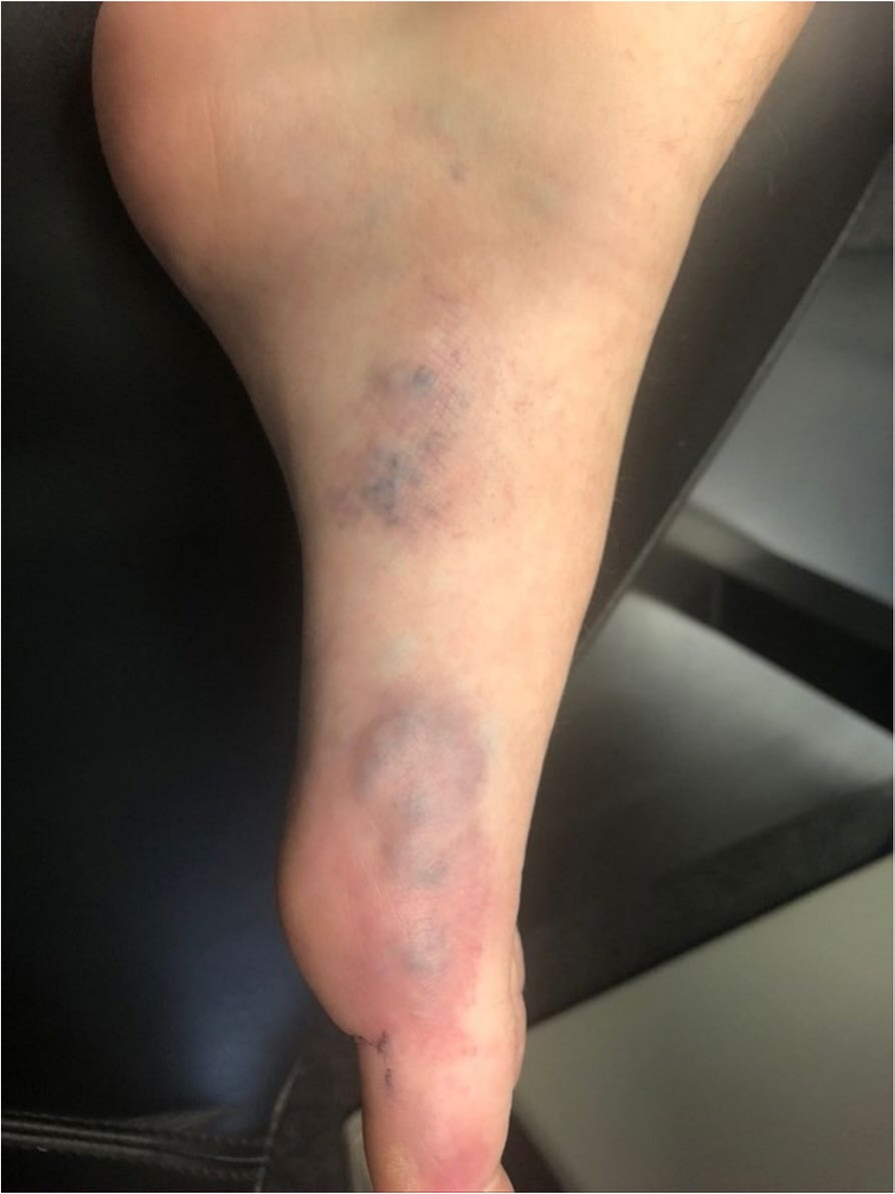
Aspect of a superficial VM on the left foot

**Fig. 2:**
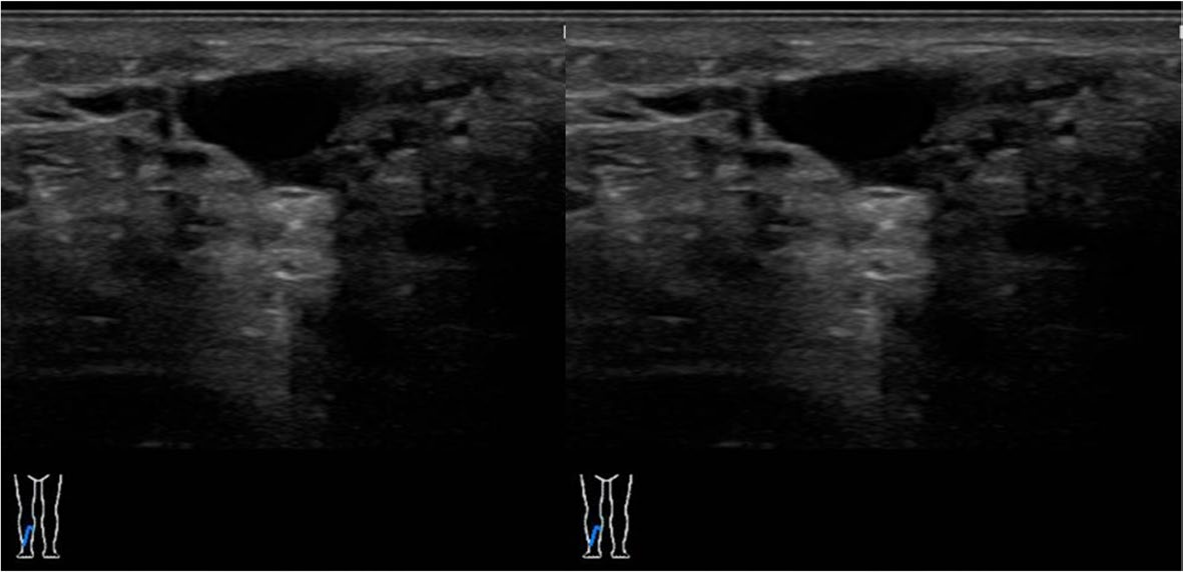
US imaging of a VM on the right lower leg in a 20-year-old patient

**Fig. 3:**
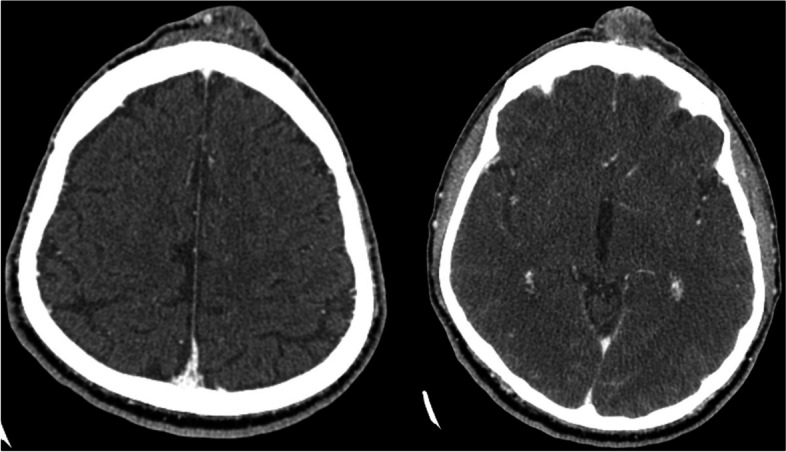
Imaging of a VM on the forehead of a 55-year-old patient using CT

**Fig. 4:**
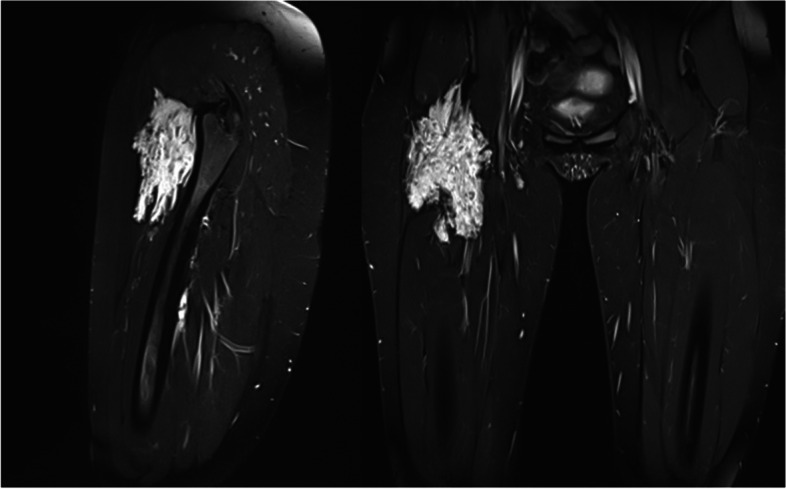
Imaging of an extensive VM on the right thigh in a 24-year-old patient

**Fig. 5:**
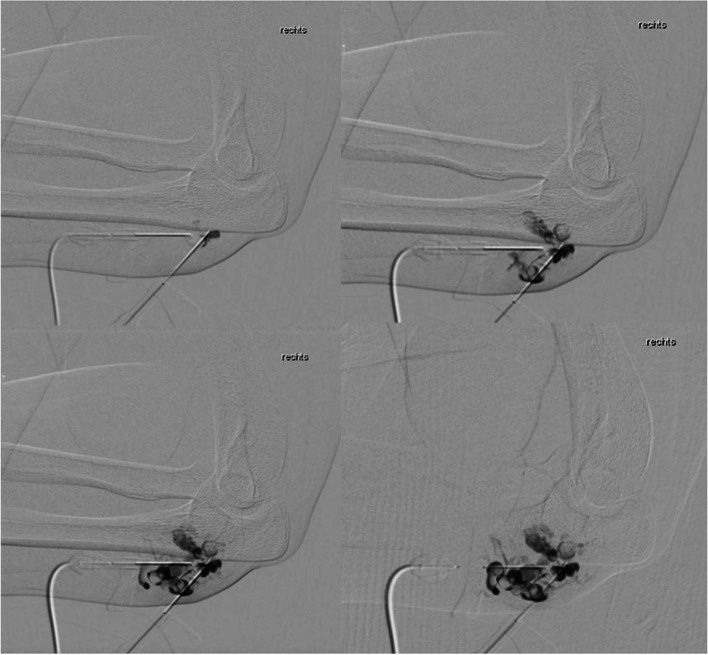
Slow filling of a VM of the right elbow after contrast medium injection

**Fig. 6:**
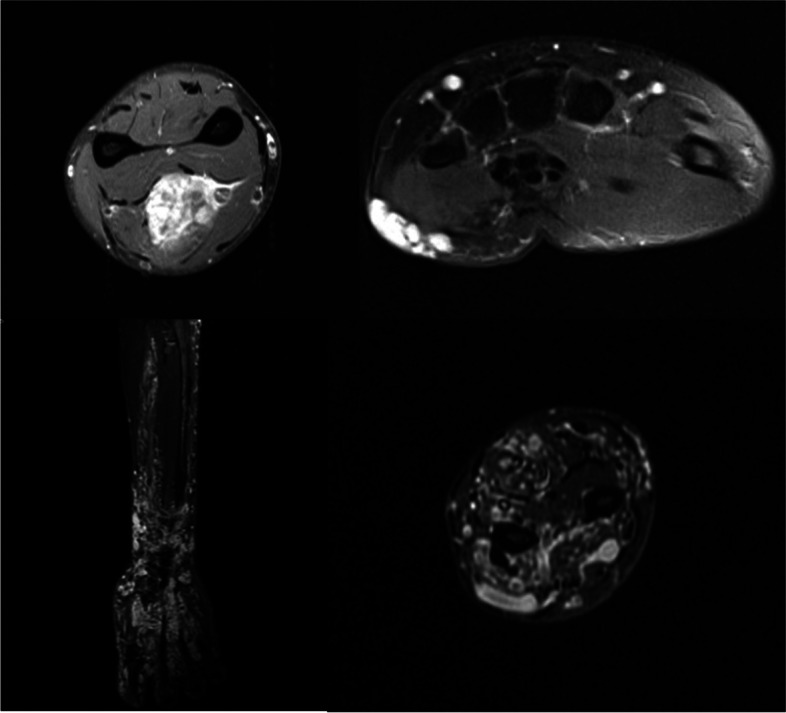
Aspect of a well-defined (top) and diffuse (bottom) VM on MRI

**Fig. 7:**
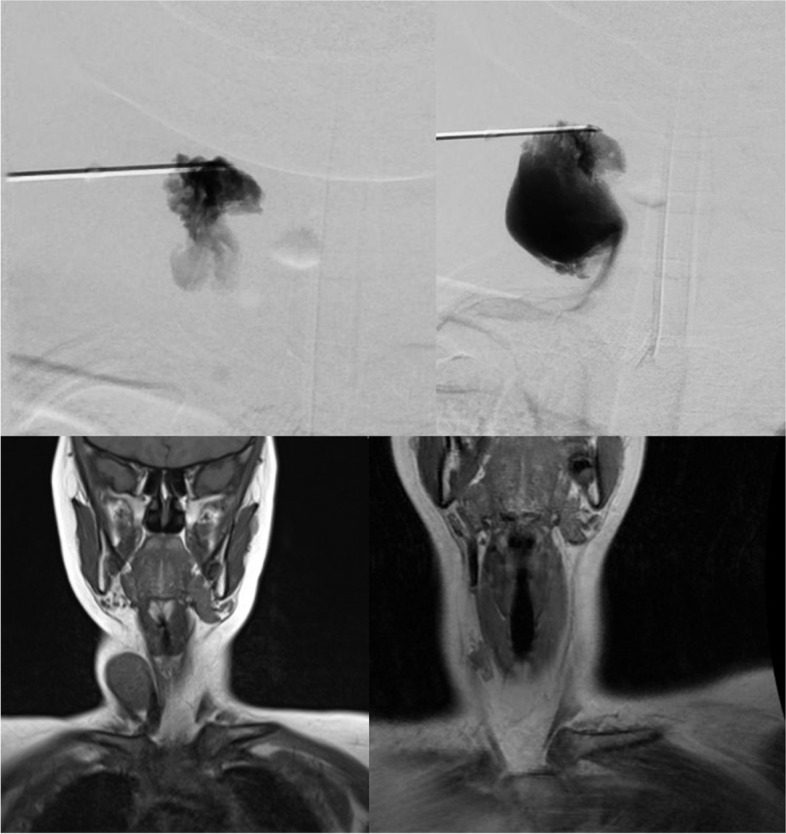
Patient with a VM in the neck area. Above: Phlebographic findings before the intervention. Bottom: MRI findings before the intervention (left) and after (right)

Almost all patients (94.1%, *n* = 16) with symptom relief were satisfied with the procedure, while this was the case for only one person without symptom reduction. A positive treatment result, meaning a partial or complete symptom reduction, was thus significantly associated with higher patient satisfaction (*p* = 0.001).

(Tables 1, 2, 3, 4, 5, 6, 7, 8, 9, 10, 11, 12, 13 and 14) (Figs. 1, 2, 3, 4, 5, 6 and 7).

**Table 1:** Hamburg Classification of vascular malformations (1988)

Predominant type	Lesion form	
	Truncular	Extratruncular
predominantly arterial	aplasia or obstruction dilatation	infiltrative limited
predominantly venous	aplasia or obstruction dilatation	infiltrative limited
predominantly lymphatic	aplasia or obstruction dilatation	infiltrative limited
arteriovenous shunt	deep superficial	infiltrative limited
combined malformations	arterial and venous without shunt	haemolymphatic infiltrative or limited
	haemolymphatic with or without shunt

**Table 2:** ISSVA Classification of vascular anomalies

Vascular tumors	Vascular malformations		
	Simple	Combined	Associated with other anomalies
• benign • locally aggressive or borderline • malignant	slow-flow: • LMs • VMs • CMs	• CVMs • CLMs • CVLMs	• Sturge-Weber • Proteus • others
	fast-flow: • AVMs • AV fistula	• CAVMs • CAVLMs	• Parkes-Weber • CLOVES • others

**Table 3:** Schobinger staging system for AVMs

Stage	Symptome
stage I	quiescence: cutaneous blush/ warmth
stage II	expansion: enlargement, pulsation, bruit
stage III	destruction: dystrophic skin changes, ulceration
stage IV	decompensation: cardiac failure

**Table 4:** ISSVA Classification of vascular tumors

Benign	Lokally aggressive	Malignant
infantile hemangioma	kaposiform hemangioendothelioma	angiosarcoma
congenital hemangioma	retiform hemangioendothelioma	epitheloid hemangioendothelioma
epitheloid hemangioma	kaposi sarcoma	others
tufted angioma	PILA + dabska tumor	
spindle-cell hemangioma	composit hemangioendothelioma	
others	others

**Table 5:** Diagnostic tools before and during treatment

Diagnostic tools	Number (n)	Share (%)
**clinical examination**	28	100
**MRI**	26	92,9
*external*	6	21,4
*internal*	20	71,4
**CT**	8	28,6
*external*	5	17,9
*internal*	3	10,7
**US**^**a**^	10	35,7
**US**^**b**^	28	100
**Phlebography**^**b**^	26	92,9

^a^ before intervention

^b^during intervention

**Table 6:** Imaging features of sfMs

	Number (n)	Share (%)
**infiltration (LMs und VMs, *n* = 28)**		
*cutaneus/subcutaneus*	17	60,7
*bone*	20	71,4
*muscle*	6	21,4
*other organs*	0	0
**margin (VMs, *n* = 25)**		
*infiltrating*	15	60
*limited*	10	40
**size (LMs, *n* = 3)**		
*macrocystic*	3	100
*microcystic*	0	0
**venous drainage (VM, *n* = 25)**		
*slow*	15	60
*fast*	8	32
*unclear*	2	8

**Table 7:** Symptoms reported by patients with sfMs

Symptoms	Number (n)	Share (%)
pain	26	92,9
swelling	26	92,9
cosmetic complaints	18	64,3
functional impairment	18	64,3
blood congestion	1	3,6
bleeding	1	3,6
respiratory distress	1	3,6
pressure	1	3,6

**Table 8:** Change in major symptoms after Sclerotherapy

	Complete relief	Partial relief	No change	Increase	Unclear
pain (*n* = 26)	9 (34,6%)	6 (23,1%)	5 (19,2%)	1 (3,8%)	5 (19,2%)
swelling (*n* = 26)	9 (34,6%)	8 (30,8%)	6 (23,1%)	0 (0%)	3 (11,5%)
cosmetic complaints (*n* = 18)	4 (22,2%)	4 (22,2%)	8 (44,4%)	1 (5,6%)	1 (5,6%)
functional impairment (*n* = 18)	6 (33,3%)	5 (27,8%)	6 (33,3%)	1 (5,6%)	0 (0%)

**Table 9:** Potential indicators for therapeutic outcome

	**Therapeutic success**		*p*-value*
	Yes	No	
**bone infiltaration** (*n* = 26)			
yes	4	2	0,60
no	16	4	
**muscle infiltration** (*n* = 26)			
yes	15	3	0,33
no	5	3	
**venous drainage** (*n* = 23)			
fast	6	2	0,59
slow	13	2	
**volume** (*n* = 19)			
< 250 ml	9	1	1
> 250 ml	8	1	

^*^Fisher`s exact test

**Table 10:** Share of certain complications after treatment

Complications	Number (n)	Share (%)
pain	6	23,1
swelling	5	19,2
inflammation	1	3,8
neurol. complications	1	3,8
bleeding	1	3,8
others	3	11,5
no complications reported	14	53,8

**Table 11:** Severity of certain complications (Society for Interventional Radiology Guidelines)

	A	B	C	D	E	F
pain (*n* = 6)	5	1				
swelling (*n* = 4)	3		1			
inflammation (*n* = 1)	1					
neurol. complications (*n* = 1)	1					
bleeding (*n* = 1)	1					
others (*n* = 3)	1	1		1

**Table 12:** Potential indicators for complications during treatment

	**Complications**		*p*-value*
	Yes	No	
**bone infiltration** (*n* = 26)			
yes	4	2	0,37
no	8	12	
**muscle infiltration** (*n* = 26)			
yes	8	11	0,67
no	4	3	
**venous drainage** (*n* = 23)			
fast	4	4	0,69
slow	6	9	
**volume** (*n* = 19)			
< 250 ml	4	6	1
> 250 ml	4	5	
**number of treatment sessions** (*n* = 26)			
one session	6	11	0,22
more than one session	6	3	

^*^Fisher`s exact test

**Table 13:** Potential indicators for patient satisfaction

	**Patient**		*p*-value*
	Satified	Unsatisfied	
**treatment outcome** (***n* = 23)**			
symptom relief	16	1	** < 0,01**
no symptom relief	1	5	
**bone infiltration** (***n* = 21)**			
yes	3	2	0,60
no	12	4	
**muscle infiltration** (***n* = 21)**			
yes	11	5	1
no	4	1	
**venous drainage** (***n* = 21)**			
fast	5	3	0,63
slow	10	3	
**volume** (***n* = 17)**			
< 250 ml	7	2	1
> 250 ml	7	1	
**number of treatment sessions** (***n* = 21)**			
one session	9	5	0,61
more than one session	6	1	

^*^Fisher`s exact test

**Table 14:** Important studies concerning the treatment of sfM

Author	Publication date	sfMs type	Number of patients	Sclerosing agent	Location
*van der Linden* et al	2009	VMs	66	Polidocanol Ethanol	all peripheral VMs
*Zhan *et al	2020	VMs	38	Polidocanol	peripheral limb VMs
*Nakamura *et al	2014	VMs	40	Polidocanol Ethanol EO	peripheral limb VMs
*Gorman *et al	2018	VMs	34	Ethanol STS	all peripheral VMs
*Teusch *et al	2016	VMs	31	Ethanol	all peripheral VMs
*Nevesny *et al	2021	VMs, LMs	26	Bleomycin	all peripheral VMs
*Clemens *et al	2017	VMs	77	Ethanol STS	all peripheral VMs
*Lee *et al	2003	sfMs and hfMs	87	Ethanol	all peripheral VMs

## Discussion

In this study, the focus was on long-term symptom reduction after percutaneous sclerotherapy of sfMs. Numerous studies have a similar topic, but differ significantly in their implementation. Some of the publications only consider lesions in certain anatomical regions (Marrocco-Trischitta et al. 2001) while in other publications all peripheral malformations are included. In this study all peripheral sfMs were included (…). In contrast to some large studies (Veräjänkorva et al. 2016), hfMs were not included at all. Only in our study a group of patients treated exclusively with polidocanol was investigated.

With an average of 1.5 sessions per patient (Ali and Mitchell 2017; Aronniemi et al. 2016; Behravesh et al. 2016; Brill et al. 2020), the number of interventions is lower than in many publications. However, as mentioned above, some patients were treated in other centers too.

Nevesny et al. recorded only slightly more treatment appointments (2.1 sessions) than we did (Nevesny et al. 2021). Van der Linden et al. differentiate the number of sessions according to the sclerosant used (Linden et al. 2009). The high number of interventions with polidocanol (3.1,1–8) is striking, while significantly fewer interventions were carried out with other substances (e.g. ethanol 1.2,1–5). Nakamura et al. indicate a significantly higher range of interventions (Ali and Mitchell 2017; Aronniemi et al. 2016; Behravesh et al. 2016; Brill et al. 2020; Clemens et al. 2017; Fresa et al. 2021; Gorman et al. 2018; Gulsen et al. 2011; Helm et al. 2022; Marrocco-Trischitta et al. 2001), but the average number of interventions (2.6) is in a similar range to the above-mentioned studies. A connection between patient satisfaction and the number of interventions was proven in Nakamura's publication (Nakamura et al. 2014).

The most important anatomical properties of sfMs include not only the location but also the volume. Ellipsoidal shape has been the basis for volume approximation in many studies before (e.g. Teusch et al. and Linden et al.) (Linden et al. 2009; Teusch et al. 2017). (…) Volume estimates in general have often been criticized due to the diffuse nature of most sfMs and the consequent inaccuracy of any measurements (Ali and Mitchell 2017). More complex methods might include a three-dimensional reconstruction of the sfM in question with subsequent volume calculation (…).

The measured volumes showed a wide range comparable to our results. In van der Linden et al., the average volume is 145 ml and thus markedly lower than in our study (641 ml), although there were outliers with a total volume of up to 3000 ml (Linden et al. 2009). A similar picture is drawn by Gorman et al. with an average volume of 156 ml and a range of 3-1703 ml (Gorman et al. 2018). Even larger VMs (> 4000 ml) were used in the study by Nevesny et al. (Nevesny et al. 2021).

The extent of volume reduction by sclerotherapy has only been estimated in very few studies. For example, Nevesny et al. (2021) reported a volume reduction of 2–98% for VM (*n* = 11), so in contrast to our measurements, no growth of sfM was observed after treatment at all (Nevesny et al. 2021). With LMs (*n* = 10), the result was even more pronounced with a volume reduction of over 70% in 80% of the patients. This may be due to the shorter time interval (3–6 months) between the two MRI scans. In this study the time between the initial MRI and the follow-up Imaging amounted to an average of 44,9 months (2–95 months) (Brill et al. 2020). What both studies have in common, however, is that (almost) complete obliteration of sfMs was observed in individual cases.

Our study confirmed that polidocanol is a rather complication-free sclerosant. The proportion of all patients with severe and mild complications in the patient collective examined was around 46%, comparable to other studies (van der Linden (2009): 40%, Nevesny et al.: 31%) (Nevesny et al. 2021; Linden et al. 2009). (…) Slightly higher complication rates were reported by Gulsen et al.reported (65%), with slight side effects also being the main focus here (Gulsen et al. 2011). This also agrees with the observation of van der Linden et al., according to which serious complications were not observed in patients treated with polidocanol (Linden et al. 2009).

The patients in our study most frequently initially reported symptoms such as pain, swelling or a cosmetic impairment. A comparison between the different publications shows that most symptoms respond well to sclerotherapy, at least initially. For instance, Clemens et al. report a reduction of all three major symptoms in 80% of the affected patients (Clemens et al. 2017). Especially with regard to the cosmetic result, this value is much higher than in the present work. On the other hand, a less pronounced reduction in symptoms is reported in van der Linden et al. (Linden et al. 2009). Here, only around 35% of those affected achieve an improvement in the cosmetic result. The proportion of patients with a complete or partial reduction in pain (59%) and swelling (58%) is comparable to the results of this work.

Finally, it should be noted that our study has various limitations compared to other work. The small number of patients limits statistical evaluation in general, especially subgroup analyzes are only conditionally meaningful. This may be a reason why no anatomical influencing factors on the treatment result could be identified. This turned out to be difficult even with patient collectives that were more than twice as large (Linden et al. 2009).

## Conclusion

The aim of the present study was to improve the counseling of patients with sfMs by obtaining and evaluating new information regarding symptom reduction, intervention-related complications and long-term patient satisfaction, as well as to identify possible influencing factors on the success of the treatment.

It could be shown that with an overall low complication rate, a mostly considerable reduction in symptoms could be achieved, which lasted for several years in a large proportion of the patients. Polidocanol has again proven to be an good alternative to ethanol with few side effects.

Overall sclerotherapy as a promising approach to treat sfMs. With some limitations, most patients can expect years of benefit from therapy. New therapeutic approaches, such as a combination with endovascular laser therapy, could deliver even better results in the future.

## Data Availability

All data generated or analysed during this study are included in this published article.

## Abbreviations

sfM: Slow-flow malformation
hfM: High-flow malformation
MRI: Magnetic resonance image
US: Ultrasound
CT: Computed tomography

## References

[CR1] Ali S, Mitchell SE (2017). Outcomes of Venous Malformation Sclerotherapy: A Review of Study Methodology and Long-Term Results. Semin Intervent Radiol.

[CR2] Aronniemi J, Castrén E, Lappalainen K (2016). Sclerotherapy complications of peripheral venous malformations. Phlebology.

[CR3] Behravesh S, Yakes W, Gupta N (2016). Venous malformations: clinical diagnosis and treatment. Cardiovasc Diagn Ther.

[CR4] Brill R, Deistung A, Gussew A (2020). Safety and effectiveness of percutaneous sclerotherapy for venous disorders of the labia majora in patients with vascular malformations. J Vasc Surg Venous Lymphat Disord.

[CR5] Clemens RK, Baumann F, Husmann M (2017). Percutaneous sclerotherapy for spongiform venous malformations - analysis of patient-evaluated outcome and satisfaction. Vasa.

[CR6] Fresa M, El Ezzi O, Debr A, Qanadli SD, Ney B, Mazzolai L (2021). Ultrasound-guided percutaneous endovenous laser treatment combined with sclerotherapy for the treatment of large intramuscular venous malformations. Int Angiol.

[CR7] Gorman J, Zbarsky SJ, Courtemanche RJM, Arneja JS, Heran MKS, Courtemanche DJ (2018). Image guided sclerotherapy for the treatment of venous malformations. CVIR Endovasc.

[CR8] Gulsen F, Cantasdemir M, Solak S, Gulsen G, Ozluk E, Numan F (2011). Percutaneous sclerotherapy of peripheral venous malformations in pediatric patients. Pediatr Surg Int.

[CR9] Helm M, Goldann C, Hammer S (2022). Vascular malformations of the female and male genitalia: type and distribution patterns revealed by magnetic resonance imaging. Clin Exp Dermatol.

[CR10] Marrocco-Trischitta MM, Nicodemi EM, Stillo F (2001). Sclerotherapy for venous malformations of the glans penis. Urology.

[CR11] Nakamura M, Osuga K, Maeda N (2014). Percutaneous sclerotherapy for venous malformations in the extremities: clinical outcomes and predictors of patient satisfaction. Springerplus.

[CR12] Nevesny F, Chevallier O, Falvo N (2021). Bleomycin for percutaneous sclerotherapy of venous and lymphatic malformations: a retrospective study of safety, efficacy and mid-term outcomes in 26 patients. J Clin Med.

[CR13] Odeyinde SO, Kangesu L, Badran M (2013). Sclerotherapy for vascular malformations: complications and a review of techniques to avoid them. J Plast Reconstr Aesthet Surg.

[CR14] Schmidt VF, Masthoff M, Goldann C (2021). Percutaneous Sclerotherapy of Venous Malformations of the Hand: A Multicenter Analysis. Cardiovasc Intervent Radiol.

[CR15] Schmidt VF, Masthoff M, Brill R (2022). Image-Guided Embolotherapy of Arteriovenous Malformations of the Face. Cardiovasc Intervent Radiol.

[CR16] Spence J, Krings T, TerBrugge KG, Agid R (2011). Percutaneous treatment of facial venous malformations: a matched comparison of alcohol and bleomycin sclerotherapy. Head Neck.

[CR17] Teusch VI, Wohlgemuth WA, Hammer S (2017). Ethanol-Gel Sclerotherapy of Venous Malformations: Effectiveness and Safety. AJR Am J Roentgenol.

[CR18] van der Linden E, Pattynama PM, Heeres BC, de Jong SC, Hop WC, Kroft LJ (2009). Long-term patient satisfaction after percutaneous treatment of peripheral vascular malformations. Radiology.

[CR19] Veräjänkorva E, Rautio R, Giordano S, Koskivuo I, Savolainen O (2016). The Efficiency of Sclerotherapy in the Treatment of Vascular Malformations: A Retrospective Study of 63 Patients. Plast Surg Int.

[CR20] Yakes WF, Haas DK, Parker SH (1989). Symptomatic vascular malformations: ethanol embolotherapy. Radiology.

